# Metformin improves the angiogenic functions of endothelial progenitor cells via activating AMPK/eNOS pathway in diabetic mice

**DOI:** 10.1186/s12933-016-0408-3

**Published:** 2016-06-18

**Authors:** Jia-Wen Yu, Ya-Ping Deng, Xue Han, Guo-Fei Ren, Jian Cai, Guo-Jun Jiang

**Affiliations:** Department of Pharmacy, Zhejiang Xiaoshan Hospital, Hangzhou, 311202 Zhejiang China

**Keywords:** Metformin, Endothelial progenitor cells (EPCs), Angiogenesis, Diabetes mellitus

## Abstract

**Background:**

Endothelial dysfunction has been suggested as a possible causal link between hyperglycemia and microvascular complications in diabetes mellitus. The effect of metformin on endothelial progenitor cells (EPCs) is still unclear. This study was designed to test the hypothesis that metformin could accelerate wound healing by improving the impaired EPC functions in streptozotocin-induced diabetic mice.

**Methods:**

Streptozotocin (STZ, 60 mg/kg/d × 5 d, *i.p.*) was injected to induce type 1 diabetes in male C57BL/6 mice. Mice were treated with metformin (250 mg/kg/d, *i.g.*) for consecutive 14 days. Wound closure was evaluated by wound area and number of CD31 stained capillaries. Functions of bone marrow-endothelial progenitor cells (BM-EPCs) were assessed by tube formation and migration assays, and expression of AMP-activated protein kinase (AMPK) and endothelial nitric oxide synthase (eNOS) was determined by western blot analysis.

**Results:**

Metformin accelerated wound closure and stimulated angiogenesis in diabetic mice. The number of circulating EPCs was increased significantly in metformin treated diabetic mice. Abilities of tube formation and migration of BM-EPCs were impaired in diabetic mice, which were improved by metformin. Expression of both phosphorylated-AMPK and phosphorylated-eNOS was significantly increased, and nitric oxide (NO) production was enhanced by metformin in BM-EPCs of diabetic mice. In vitro, metformin improved impaired BM-EPC functions, and increased phosphorylated-eNOS expression and NO production in cultured BM-EPCs caused by high glucose, which was prevented by the AMPK inhibitor compound C.

**Conclusions:**

Our results suggest that metformin could improve BM-EPC functions in STZ-induced diabetic mice, which was possibly dependent on the AMPK/eNOS pathway.

**Electronic supplementary material:**

The online version of this article (doi:10.1186/s12933-016-0408-3) contains supplementary material, which is available to authorized users.

## Background

Diabetes mellitus is the most serious, chronic metabolic disorder, which is characterized by hyperglycemia, and affecting more than 400 million people worldwide with a predicted >50 % increase in 2030 [1, 2]. In particular, microvascular and macrovascular disease, one of the main complications of diabetes, has been proposed as an important risk factor for the death of diabetic patients [3, 4].

Endothelial dysfunction is not only strongly associated with risk of vascular disease, but also the inner cause of impaired wound healing ability [5, 6]. Endothelial progenitor cells (EPCs) play a primary role in angiogenesis but are functionally impaired in diabetes, which may contribute to endothelial dysfunction [7]. EPCs are vital for the vascular protection and angiogenesis [8]. In patients with diabetes, circulating EPCs number is decreased and EPC functions is impaired [9, 10], which are believed to be one of the pathogenesis of vascular complications in diabetes, as manifested by impaired wound healing.

Metformin is a biguanide derivative, which is the most widely prescribed oral hypoglycaemic drug for the management of type 2 diabetes mellitus [11, 12]. Several studies have focused on the effects of metformin on EPCs in patients with diabetes, and found that patients with type 2 diabetes treated with metformin had significantly increased number of circulating EPCs [13]. Recent studies demonstrated that activation of AMP-activated protein kinase (AMPK) partly mediated the therapeutic effects of metformin, which stimulated ischemia-induced revascularization in the ischemic hindlimb mice model [14–16]. Furthermore, studies found that clinically relevant concentrations of metformin activated AMPK in cultured endothelial cells (ECs) [17–21]. AMPK is a well conserved heterotrimer protein comprising α, β, and γ subunits, each of which has 2 or 3 genes encoding [22]. Findings support the notion that AMPK was a new regulator of angiogenesis, which was specifically required for ECs migration and differentiation under hypoxia conditions [23, 24]. In addition, it was found that activated AMPK could directly phosphorylate endothelial nitric oxide synthase (eNOS) in ECs and promote endothelial function [25–28]. However, little information exists regarding the role of metformin on EPC functions under diabetic conditions. Thus, our study tested the hypothesis that metformin could accelerate wound healing, at least in part, by improving the angiogenic functions of EPCs with an AMPK related pathway in diabetic mice.

## Methods

### Animals and treatments

**Fig. 1:**
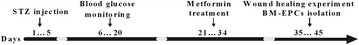
Illustration of experimental protocols. STZ (60 mg/kg/d × 5 d, *i.p.*) was given to induce diabetes in C57BL/6 mice, and blood glucose was monitored. Metformin (250 mg/kg/d × 14 d, *i.g.*) was given for consecutive 14 days. Then wound healing, BM-EPC functions and proteins were measured

Male C57BL/6 mice (6w, 18–20 g), purchased from the Sino-British SIPPR/BK Lab Animal Ltd (Shanghai, China), were housed in controlled conditions (temperature: 23 ± 2 °C; lighting: 8:00–20:00) and received a standard mouse chow and tap water ad libitum. All the animals used in this work received humane care in compliance with the institutional animal care guidelines and the Guide for Care and Use of Laboratory Animals published by the National Institutes of Health. Mice were injected with streptozotocin (STZ; Amresco, USA; 60 mg/kg/d × 5 d, *i.p.*) dissolved in 0.1 mM sodium citrate buffer (pH 4.5). Random blood glucose was measured by using the blood glucose monitoring system (MAJOR, Taiwan) with whole blood from the mouse tail vein. On day 21, mice with the random blood glucose value ≥300 mg/dL were defined as STZ-induced diabetic mice, which were subsequently divided into two groups: treated with metformin (250 mg/kg/d × 14 d, *i.g.*, n = 26) or vehicle (0.5 % CMC-Na × 14 d, *i.g.*, n = 26); age-matched mice without STZ treatment served as the control also received vehicle (0.5 % CMC-Na × 14 d, *i.g.*, n = 26). On day 35, mice were used for wound healing experiment, or anesthetized to harvest bone marrow to isolate EPCs (Fig. 1).

### Evaluation of wound healing and angiogenesis

Mice were anesthetized with ketamine (100 mg/kg, *i.p.*), and fixed to remove hair on the dorsum by swabbing with betadine and 75 % ethanol before wounding [29]. A 6 mm circular wound was made by punch biopsy, and closure of the wounded area was measured every 2 days until day 10. The wound area was digitized and areas were calculated.

Skin at the wounded area was harvested on day 3, 6 and 9 after punch biopsy. Evaluation of angiogenesis was conducted by CD31 immunochemistry and hematoxylin staining [29, 30]. Capillaries were recognized as tubular structures positive for CD31. One slide from each mouse was examined under high-power fields.

### Determination of circulating EPCs

About 0.5 ml blood was harvested from anesthetized mice, and were dissolved in PBS (1:1). The samples were added into 1 ml gradient centrifugation liquid 10831 (Sigma, St. Louis, MO, USA) gently, and followed by 3000 rpm centrifugation for 25 min. The cell layer was extracted and underwent 5 min lysis of erythrocyte. The samples were incubated with FITC-Sca-1 (BD, San Diego, CA, USA) and PE-Flk-1 (BD, San Diego, CA, USA) antibodies for flow cytometry examination [29].

### Assessment of BM-EPC functions

BM-EPCs from C57BL/6 mice were isolated, cultured and identified as previously described [31]. To confirm the BM-EPCs phenotypes, cells were stained for the uptake of Dil-acLDL (Molecular Probes Inc., Eugene, OR, USA) and FITC-labeled Ulex europaeus agglutinin (lectin; Sigma-Aldrich, St. Louis, MO, USA). The abilities of tube formation and migration of BM-EPCs were determined to assess EPC functions. The angiogenic capacity of BM-EPCs was determined by Matrigel tube formation assay. Briefly, BM-EPCs were isolated and cultured for 7 days, and were harvested using 0.125 % trypsin. The cells with a concentration of 5 × 10^4^/100 μl were plated to a 96-well plate pre-coated with 50 μl/well growth factor-induced Matrigel (BD Biosciences, Bedford, MA, USA). After 6 h incubation at 37 °C, tube formation ability of BM-EPCs was evaluated by counting tube numbers. Images of tube morphology were taken under the inverted phase contrast microscope.

The migratory ability of EPCs was assessed by a modified Boyden chamber assay. The cells with a concentration of 5 × 10^4^/100 μl were plated on the upper chamber with polycarbonate membrane (8 μm pores), and VEGF (50 ng/ml) was added to the cell-free medium in lower chamber of a 24-well Transwell plate (Corning Transwell, Lowell, MA, USA). After incubation at 37 °C for 24 h, cells were fixed with 2 % paraformaldehyde, and stained by Hoechst 33258 (10 μg/ml). The migrated cells were observed under the fluorescence microscope.

### Determination of SDF-1α protein by ELISA

The serum concentration of SDF-1α protein in mice after metformin treatment was determined by ELISA (R&D systems, Minneapolis, MN, USA) according to manufacturer’s instruction.

### In vitro study

#### Evaluation of BM-EPC functions

BM-EPCs were isolated from male C57BL/6 mice, and cultured for 7 days. Medium were replaced with high glucose (HG, 33 mM, M199 containing 27.5 mM d-glucose) medium or high glucose medium containing metformin (2 mM) for 24 h [32]. The basal M199 containing 5.5 mM glucose (normal glucose, NG) was served as control, and M199 containing 27.5 mM mannitol was used as an osmotic control (Mtol). In addition, metformin (2 mM) or compound C (10 μM) was added into the medium alone and maintained for 24 h to see the basal effects of the compounds. Effects of metformin on high glucose induced EPCs dysfunction were assessed by functional analysis.

#### Measurement of AMPK and eNOS expression

The samples were prepared as described previously [33]. Protein concentrations were quantified by the BCA Protein Assay Kit (Thermo, Rockford, USA). Samples containing equal amounts of protein were subjected to SDS-PAGE in a Bio-Rad miniature slab gel apparatus and electrophoretically transferred onto a nitrocellulose membrane. The membranes were incubated with 5 % BSA/PBST for 1 h at 25 °C, and incubated overnight at 4 °C with primary antibodies and 1 h at 25 °C with secondary antibodies respectively. Cell Signaling Technology Inc (Danvers, Mass) was the supplier for the following primary antibodies: rabbit polyclonal anti-phospho-eNOS (Ser1177) antibody, rabbit anti-phospho-AMPK (Thr172) antibody, rabbit anti-AMPK antibody. Mouse anti-eNOS monoclonal antibody was obtained from BD Transduction Laboratories (Lexington, Ky).

#### Detection of intracellular NO

Intracellular NO level was detected by membrane-permeable probes DAF-FM diacetate (Invitrogen, Carlsbad, CA). Briefly, 7 days after BM-EPCs isolation, the cells were harvested using 0.125 % trypsin. After suspension, the samples were incubated with DAF-FM diacetate (10^−6^ mol/L) for 30 min at 37 °C and 30 min at room temperature in dark respectively for flow cytometry examination.

### Statistical analysis

Data are presented as mean ± SEM. Statistical analysis was performed by one-way analysis of variance (ANOVA) with Newman–Keuls multiple comparison test. A value of *P* < 0.05 was considered to be statistically significant.

## Results

### Body weight and blood glucose changes in diabetic mice

**Fig. 2:**
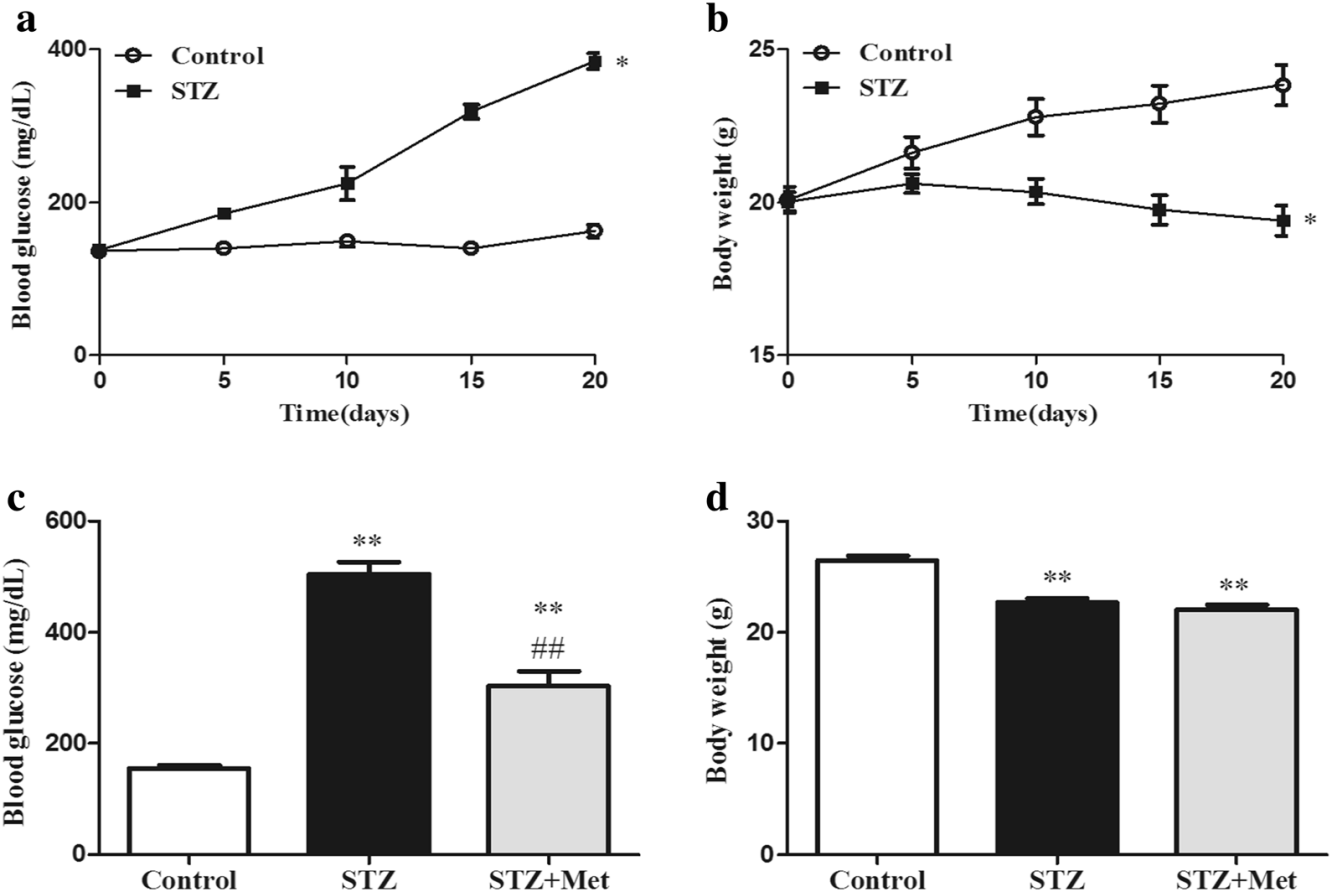
Establishment of STZ-induced diabetic mice. Compared with the control, mice treated with STZ (60 mg/kg/d × 5 d, *i.p.*) displayed higher blood glucose (**a**) and lower body weight (**b**). Metformin (250 mg/kg/d × 14 d, *i.g.*) decreased blood glucose (**c**), but did not change the body weight (**d**) in STZ-induced diabetic mice. **P < 0.01, *P < 0.05 *vs* Control; ^*##*^ *P* < 0.01 vs STZ. Values are mean ± SEM (n = 10 per group)

Blood glucose in STZ-induced diabetic mice was significantly increased (384 ± 10 vs 162 ± 8 mg/dl, *P* < 0.05; Fig. 2a), while body weight was significantly decreased when compared with the control after STZ injection on day 20 (19 ± 0.4 vs 24 ± 0.6 g, *P* < 0.05; Fig. 2b). Metformin treatment for consecutive 14 days significantly decreased the blood glucose level, but did not modify the body weight in STZ-induced diabetic mice (*P* < 0.01; Fig. 2c, d).

### Metformin accelerated wound closure and angiogenesis in diabetic mice

**Fig. 3:**
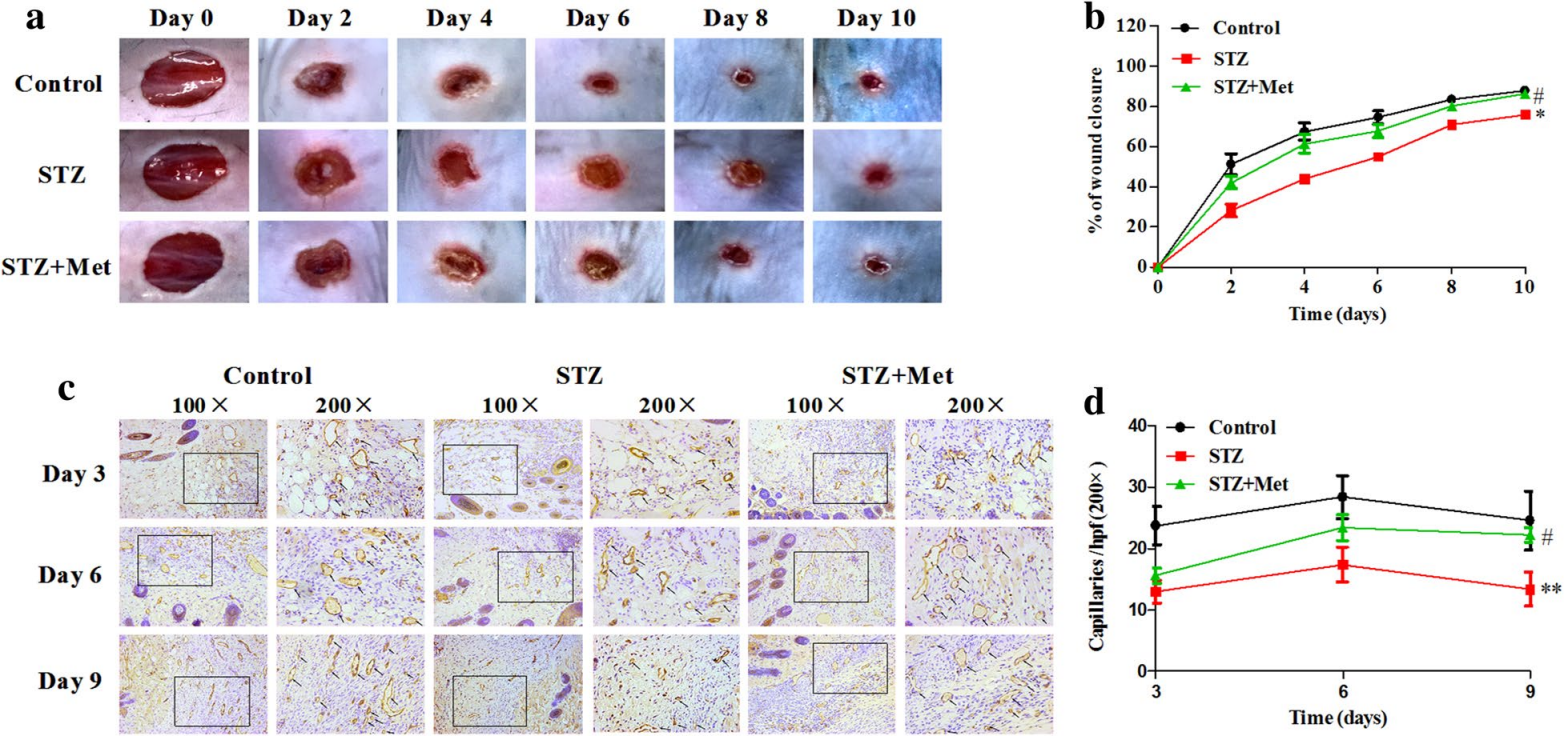
Metformin accelerated wound healing and stimulated angiogenesis in diabetic mice. Six-mm diameter wound was made by punch biopsy and closure of the wound area was measured every 2 days until day 10, metformin accelerated wound closure in STZ-induced diabetic mice (**a**, **b**). Typical photographs of CD31-positive staining on the wounded area were obtained on day 3, 6 and 9 (**c**,** d**). **P < 0.01, *P < 0.05 vs Control; ^*#*^ *P* < 0.05 vs STZ. Values are mean ± SEM (n = 5 per group)

To assess the effects of metformin on wound healing in STZ-induced diabetic mice, the percentage of wound closure was measured every other day until day 10. Wound healing was significantly slowed in STZ-induced diabetic mice when compared to the control, and metformin significantly accelerated the wound closure (*P* < 0.05; Fig. 3a, b) in diabetic mice.

The number of CD31-positive tubular structures in the wounds and surrounding skin were calculated to further evaluate the role of metformin on neovascularization. Capillary formation in STZ-induced diabetic mice was significantly worse on day 3, 6 and 9 when compared to control. However, metformin significantly improved the capillary formation in diabetic mice on day 6 and 9 (*P* < 0.05, Fig. 3c, d).

### Metformin improved BM-EPC functions in diabetic mice

**Fig. 4:**
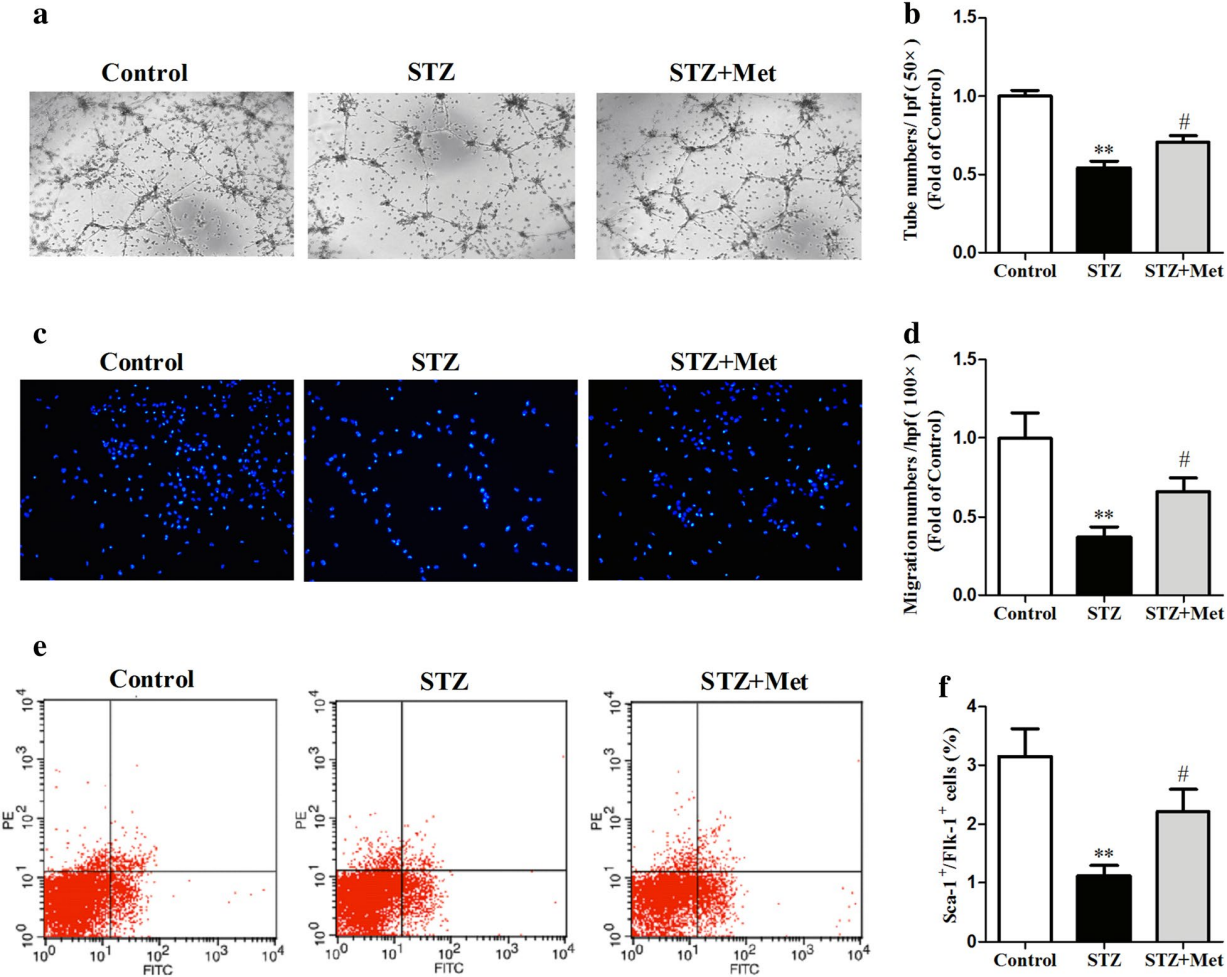
Metformin improved BM-EPC functions in diabetic mice. BM-EPCs from anesthetized mice were isolated and cultured. Metfromin improved the tube formation (**a**, **b**) and migration (**c**, **d**) ability of BM-EPCs. Blood was harvested from anesthetized mice. Metformin increased circulating EPCs in STZ-induced diabetic mice (**e**, **f**). **P < 0.01 vs Control; ^*#*^ *P* < 0.05 vs STZ. Values are mean ± SEM (n = 6 per group)

To test that metformin increased the capillary densities by improving the angiogenic functions of EPCs in STZ-induced diabetic mice, the capacities of tube formation and migration of BM-EPCs were assessed. It was found that functions of BM-EPCs in diabetic mice were significantly lower compared to control. Metformin significantly increased both the capacities of tube formation (0.70 ± 0.04 vs 0.54 ± 0.04, *P* < 0.05; Fig. 4a, b) and migration (0.66 ± 0.08 vs 0.37 ± 0.06, *P* < 0.05; Fig. 4c, d) in STZ-induced diabetic mice. Moreover, metformin significantly increased circulating EPCs in STZ-induced diabetic mice (2.18 ± 0.32 vs 1.11 ± 0.18 %, *P* < 0.05; Fig. 4e, f). In addition, we found that the serum concentration of SDF-1α protein was not changed after metformin treatment (Additional file 1: Figure S1).

### Metformin alleviated high glucose-induced dysfunction of BM-EPCs in vitro

**Fig. 5:**
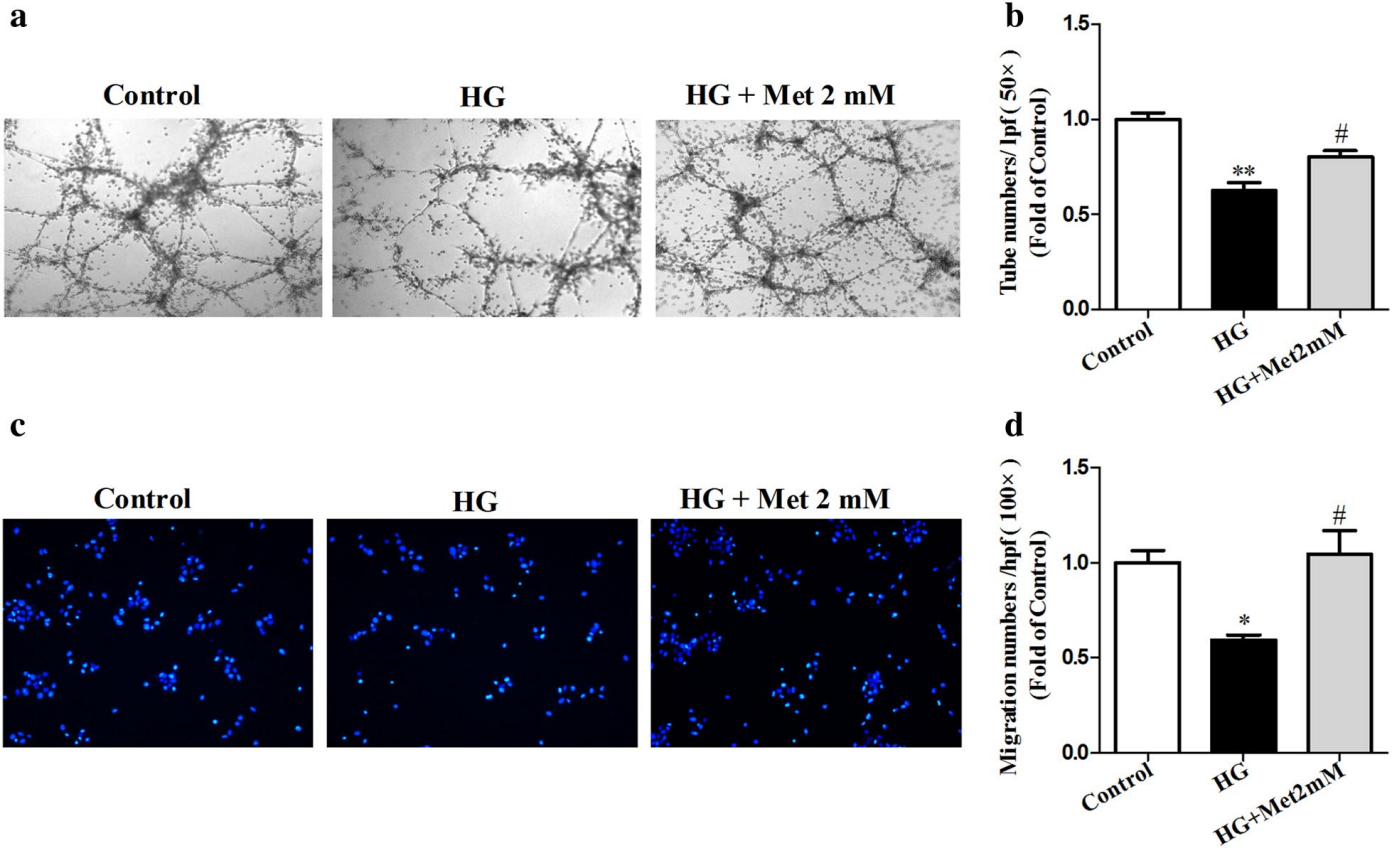
Metformin alleviated high glucose-induced dysfunction of BM-EPCs in vitro. BM-EPCs were incubated for 24 h with high glucose (33 mM), or high glucose (33 mM) + metformin (2 mM). BM-EPCs without high glucose and drug treatment were served as control. Metformin significantly increased high glucose-impaired tube formation (**a**, **b**), and migration (**c**, **d**) ability in cultured BM-EPCs in vitro. **P < 0.01, *P < 0.05 vs Control; ^*#*^ *P* < 0.05 vs HG. Values are mean ± SEM (n = 5 per group)

BM-EPCs from C57BL/6 mice were identified as Dil-acLDL and lectin double-positive cells under a fluorescence microscope (Additional file 2: Figure S2). To investigate whether the effect of metformin on EPCs in diabetic mice was directly related to the high glucose, high glucose was used to induce BM-EPC dysfunction in vitro. High glucose significantly decreased both the capacities of tube formation and migration in cultured BM-EPCs compared to the control. Metformin improved impaired BM-EPC functions caused by high glucose (Tube formation: 0.80 ± 0.03 vs 0.62 ± 0.03; Migration: 1.04 ± 0.12 vs 0.59 ± 0.03, *P* < 0.05; Fig. 5). However, compared with the NG, BM-EPCs treated with mannitol (served as the osmotic control for the HG) showed no significant changes in tube formation and migration (Additional file 3: Figure S3).

### Metformin increased both AMPK and eNOS phosphorylated-to-total ratio in BM-EPCs in diabetic mice

**Fig. 6:**
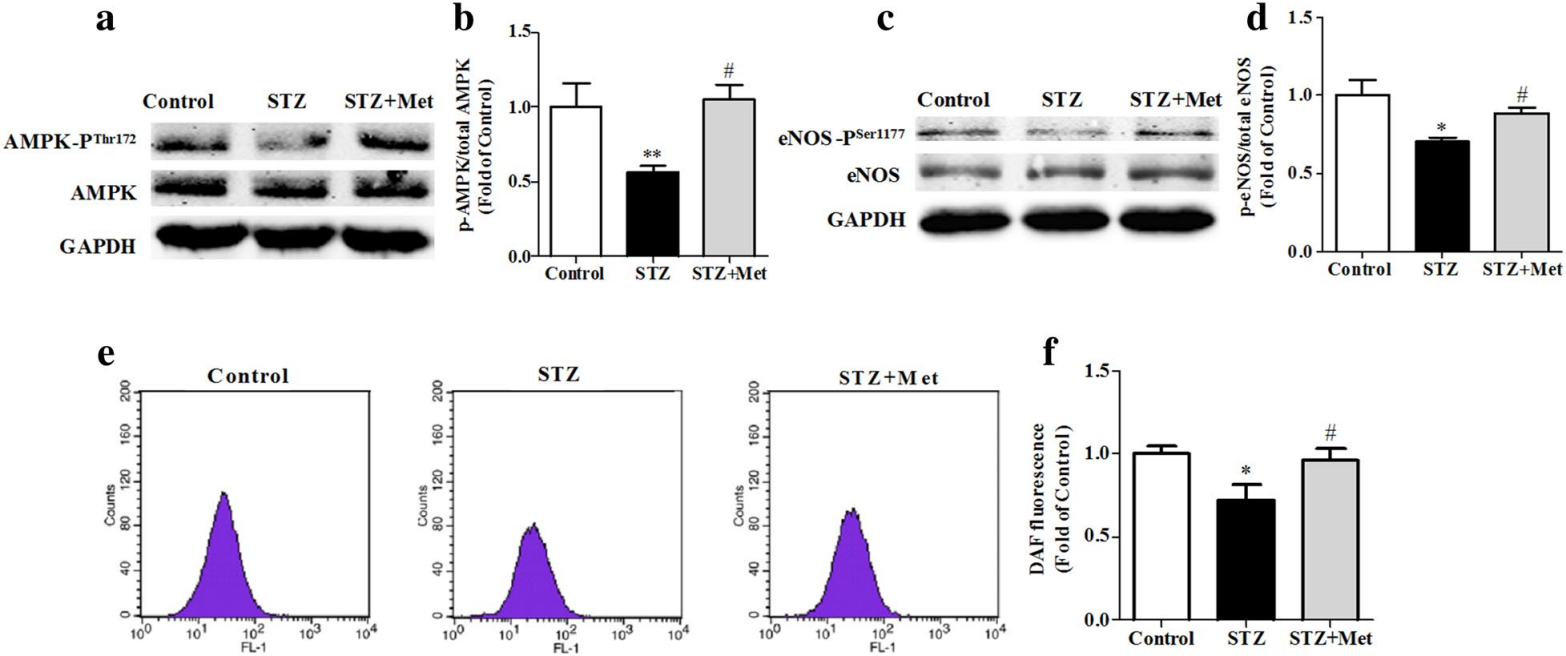
Metformin improved the impaired BM-EPCs by activating AMPK/eNOS pathway in diabetic mice. BM-EPCs from anesthetized mice were isolated and cultured. AMPK and eNOS in BM-EPCs, and intracellular NO levels were measured. Metformin significantly increased AMPK (**a**, **b**) and eNOS (**c**, **d**) phosphorylated-to-total ratio, and enhanced NO production (**e**, **f**) of BM-EPCs from diabetic mice. **P < 0.01, *P < 0.05 vs Control; ^*#*^ *P* < 0.05 vs STZ. Values are mean ± SEM (**a**, **b**, **c**, **d**: n = 4 per group; **e**, **f**: n = 6 per group)

Expression of AMPK and eNOS in BM-EPCs of STZ-induced diabetic mice was determined. Western blot analysis found that total AMPK and eNOS protein in BM-EPCs showed no difference among groups. However, both phosphorylated-AMPK and phosphorylated-eNOS expression in BM-EPCs were decreased in STZ-induced diabetic mice compared to the control. Metformin increased both phosphorylated-AMPK and phosphorylated-eNOS expression in BM-EPCs of the diabetic mice (*P* < 0.05; Fig. 6). Meanwhile, it was found that the intracellular NO level in BM-EPCs was significantly decreased in STZ-induced diabetic mice, and metformin increased the intracellular NO level (*P* < 0.05; Fig. 6).

### Metformin increased phosphorylated-eNOS in BM-EPCs via an AMPK dependent pathway in vitro

**Fig. 7:**
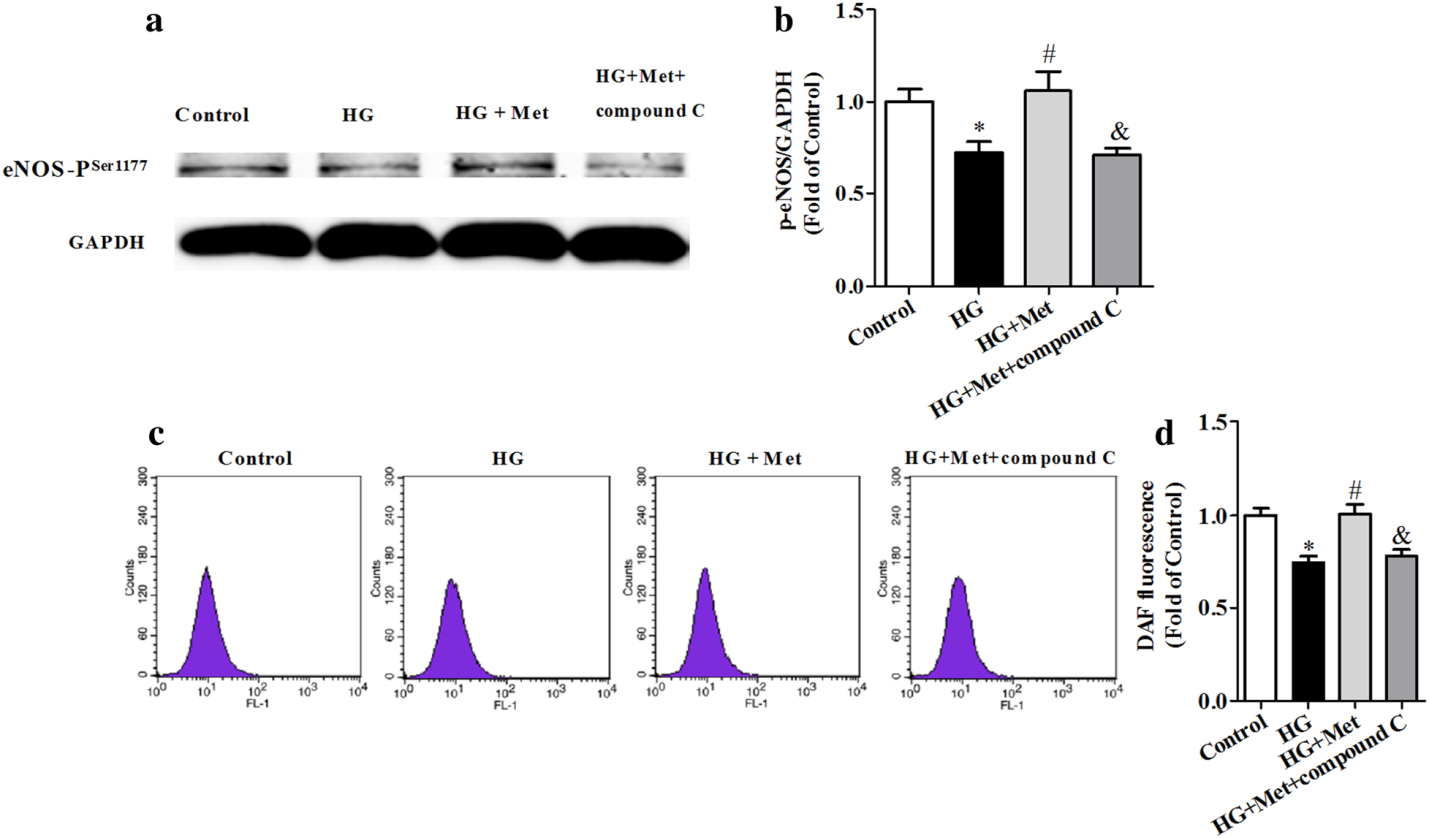
Metformin increased phosphorylated-eNOS in BM-EPCs via an AMPK dependent pathway in vitro. Metformin (2 mM) and compound C (10 μM) were added into the high glucose medium for 24 h. Metfromin significantly increased high glucose-induced phosphorylated-eNOS expression (**a**, **b**) and intracellular NO levels (**c**, **d**) in cultured BM-EPCs. Inhibition of AMPK by compound C significantly downregulated phosphorylated-eNOS expression (**a**, **b**) and NO production (**c**, **d**) enhanced by metformin in BM-EPCs cultured in vitro. *P < 0.05 vs Control; ^*#*^ *P* < 0.05 vs HG; ^&^ *P* < 0.05 vs HG + Met. Values are mean ± SEM (**a**, **b**: n = 3 per group; **c**, **d**: n = 5 per group)

To explore whether expression of phosphorylated eNOS was determined by activation of AMPK, phosphorylated-eNOS expression in BM-EPCs in vitro was measured when AMPK inhibitor was used. Similarly, high glucose induced decreased phosphorylated-eNOS in vitro, and metformin significantly prevented the change caused by high glucose. Importantly, inhibition of AMPK by compound C significantly inhibited phosphorylated-eNOS expression enhanced by metformin in BM-EPCs cultured in vitro (*P* < 0.05; Fig. 7). In addition, metformin reversed intracellular NO production caused by high glucose, which was prevented by inhibiting AMPK with compound C (*P* < 0.05; Fig. 7). Metformin alone did not change BM-EPC functions of both tube formation and migration, while compound C significantly inhibited tube formation, but not migration (Additional file 4: Figure S4).

## Discussion

In the present study, we found that: (1) metformin accelerated wound healing and stimulated angiogenesis, improved the impaired EPC capacity, and increased NO production in BM-EPCs of STZ-induced diabetic mice; (2) In vitro, metformin improved high glucose-impaired EPC function, and enhanced intracellular NO level; (3) the protective effects of metformin on BM-EPCs were possibly related to activating AMPK/eNOS pathway.

Wound healing involves an integrative interplay of cells, mediators, growth factors, and cytokines, which is responded to tissue injury [34, 35]. Under diabetic condition, impaired wound healing and reduced vascular repair may be a consequence of a metabolic derangement [34, 36, 37]. In addition, chronic hyperglycemia has been reported to be responsible for the onset of microvascular complications, including the diabetic foot ulcer and risk for amputation [38]. Angiogenesis plays a significant role in skin maintenance and repair, depending on various cell participation and coordination, especially EPCs [39, 40]. However, much of the complexity of the angiogenesis has not yet to be defined [34].

Metformin is one of the most widely used drugs for the treatment of type 2 diabetes, which improves vascular endothelial functions in type 2 diabetic patients, although its mechanisms remain largely unknown [41]. Recently, several studies showed the paradoxical effects of metformin on the angiogenesis. In general, metformin was considered to down-regulate tumor angiogenesis [42, 43]. However, in disease models such as heart failure and diabetes, metformin was associated with enhanced angiogenesis [12, 44, 45]. It was also found that metformin prevented partially insulin-deficient diabetes-induced bone microarchitecture alterations [46], and enhance the process of bone repair in diabetic and nondiabetic rats [47]. Other anti-diabetic drugs, such as dipeptidyl peptidase-4 (DPP-4) and alpha-glucosidase inhibitor could also improve endothelial function in patients with type 2 diabetes [48], rosiglitazone could improve angiogenic potential of diabetic ECs and proangiogenic cells (PACs) [49]. In our work, we found that metformin (250 mg/kg/d × 14 d, *i.g.*) did increase the capillary densities and the percentage of wound closure in STZ-induced diabetic mice. Thus, we further explored the effect of metformin on BM-EPC functions in STZ-induced diabetic mice.

Endothelial monolayer is necessary for preserving the integrity of vasculature [50]. The functional activity and even the structure of endothelial cells are impaired under diabetic conditions, which may contribute to microvascular and macrovascular abnormalities [51]. EPCs, a group of multi-progenitor cells, can differentiate into endothelial cells, and make up the lining of blood vessels [52]. EPCs are important precursors of endothelial cell, and it has been shown that EPCs were involved in angiogenesis and wound repair in diabetic patients [53–55]. Importantly, systematic administration of EPCs could significantly improve angiogenesis and wound healing in a mouse model of type 2 diabetes [56]. These suggest that improvement of EPC function or increase of circulating EPCs might be clinically significance with treatment of certain specific drugs, especially for diabetic vascular complications. It was found that the percentage of circulating EPCs and functions of BM-EPCs in diabetic mice were significantly lower compared with the control. Metformin treatment significantly prevented these changes in diabetic mice. In addition, metformin treatment improved impaired BM-EPC function caused by high glucose. Thus, we postulated that the effect of metformin in accelerating wound healing under diabetic condition was possibly related to improving impaired BM-EPC functions and increasing circulating EPCs number.

Dysfunction of EPCs contributes to the pathogenesis of diabetes, and the main cause of EPC dysfunction is the loss of protection from NO due to reduced synthesis from eNOS [57, 58]. One of the AMPK targets that may be particularly important in the circulatory system is the eNOS [59]. Moreover, AMPK was suggested as a drug target for diabetes mellitus, including drugs as metformin [60]. Activation of AMPK by metformin could protect human coronary artery endothelial cells and against diabetic lipoapoptosis [61]. In our work, metformin significantly increased both AMPK and eNOS phosphorylated-to-total ratio, and enhanced NO production of BM-EPCs from diabetic mice. In vitro, metfromin increased both phosphorylated-eNOS expression and intracellular NO levels in cultured BM-EPCs. Importantly, inhibition of AMPK by compound C prevented these changes by metformin. These indicated that the beneficial effect of metformin on BM-EPC functions in STZ-induced diabetic mice was related to activating AMPK/eNOS pathway.

## Conclusions

In summary, our work demonstrated that metformin could accelerate wound healing, stimulate angiogenesis, improve the impaired BM-EPC functions, and increase both phosphorylated-AMPK and phosphorylated-eNOS expression in BM-EPCs from STZ-induced diabetic mice. In vitro, metformin could improve high glucose-impaired BM-EPC functions, and increase phosphorylated-eNOS expression and NO production in BM-EPCs caused by high glucose. These together suggest that metformin may improve BM-EPC functions in diabetic mice possibly with an AMPK/eNOS dependent pathway.

## Additional files

10.1186/s12933-016-0408-3 Serum concentration of SDF-1α protein in mice determined by ELISA. The serum concentration of SDF-1α protein was not changed after metformin treatment (n = 5 per group).
10.1186/s12933-016-0408-3 Characterization of mouse BM-EPCs. BM-EPCs were identified as Dil-acLDL (red) and lectin (green) double-positive cells under the fluorescence microscope. Nuclei were counterstained with Hoechst (blue). Scale bar: 50 μm.
10.1186/s12933-016-0408-3 BM-EPC functions under the osmotic pressure equal to that of high glucose (HG). Compared with the normal glucose (NG), BM-EPCs treated by mannitol to make equal osmotic pressure with HG showed no significant changes in tube formation and migration.***P* < 0.01, vs NG; ^*#*^ *P* < 0.05 *vs* HG. Values are mean ± SEM (n = 5 per group).
10.1186/s12933-016-0408-3 Metformin and compound C used alone on BM-EPC functions. Compared with the control, metformin did not change BM-EPC functions of both tube formation and migration. However, compound C significantly inhibited tube formation, but not migration. ***P* < 0.01, vs Control. Values are mean ± SEM (n = 5 per group).

## Abbreviations

EPCs: endothelial progenitor cells
BM-EPCs: bone marrow-endothelial progenitor cells
ECs: endothelial cells
AMPK: AMP-activated protein kinase
eNOS: endothelial nitric oxide synthase
NO: nitric oxide
STZ: streptozotocin
CMC-Na: sodium carboxyl methyl cellulose
Dil-acLDL: acetylated low density lipoproteinl
VEGF: vascular endothelial growth factor
SDF-1α: stromal derived factor-1 alpha
Met: metformin
HG: high glucose
NG: normal glucose
Mtol: mannitol
SDS-PAGE: sDS-polyacrylamide gel electrophoresis
BSA: bovine serum albumin
PBST: phosphate buffer saline with 0.1 % Tween-20
DPP-4: dipeptidyl peptidase-4
PACs: proangiogenic cells
lpf: low-power field
hpf: high-power field
