# Supplementary figures and images for: Metformin improves the angiogenic functions of endothelial progenitor cells via activating AMPK/eNOS pathway in diabetic mice
Source: Cardiovasc Diabetol. 2016 Jun 18;15:88. doi: 10.1186/s12933-016-0408-3 (PMC4912824; doi:10.1186/s12933-016-0408-3)

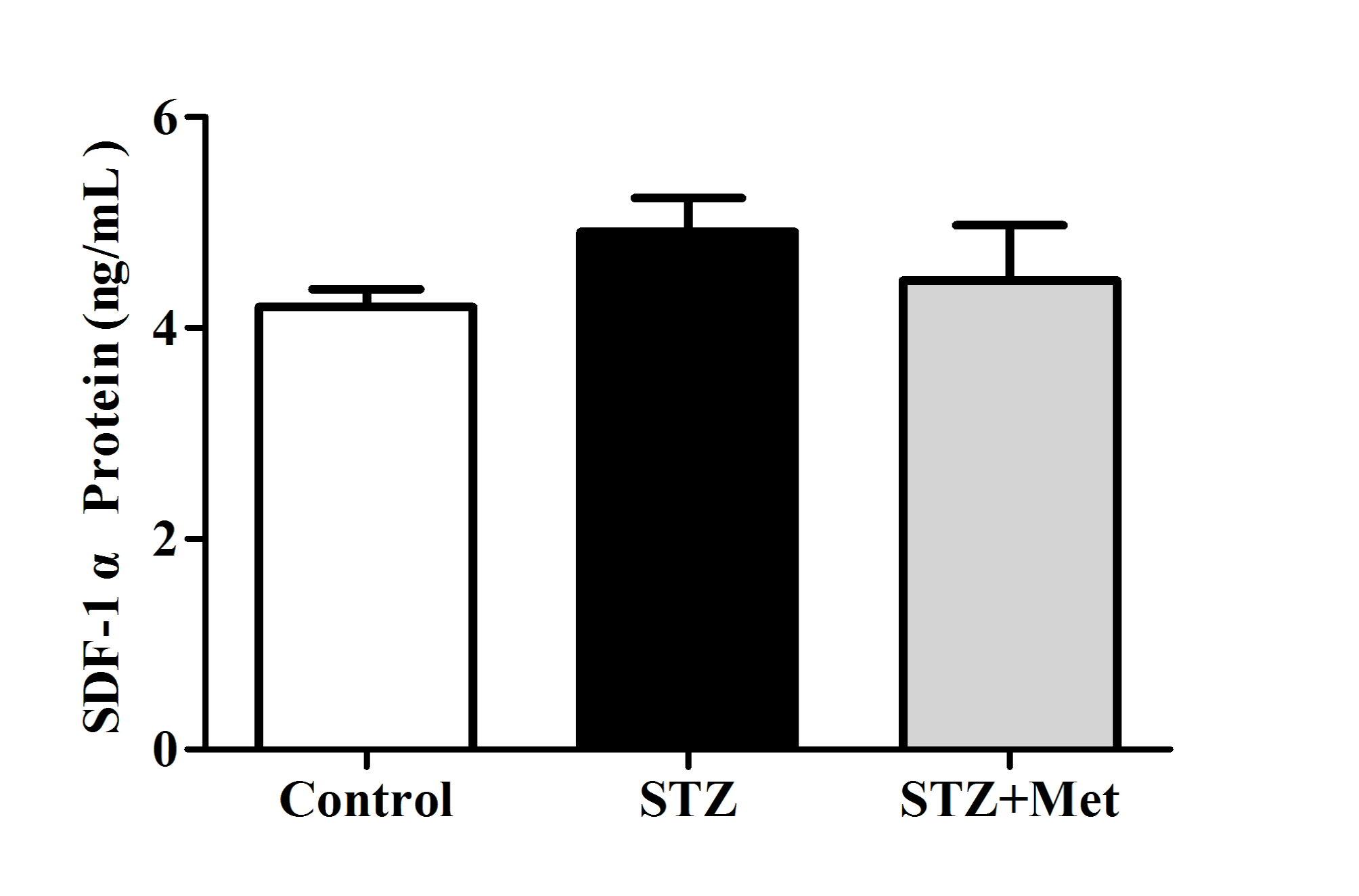

Supplement: Supplementary file 1 — 10.1186/s12933-016-0408-3 Serum concentration of SDF-1α protein in mice determined by ELISA. The serum concentration of SDF-1α protein was not changed after metformin treatment (n = 5 per group). [file 12933_2016_408_MOESM1_ESM.tif]

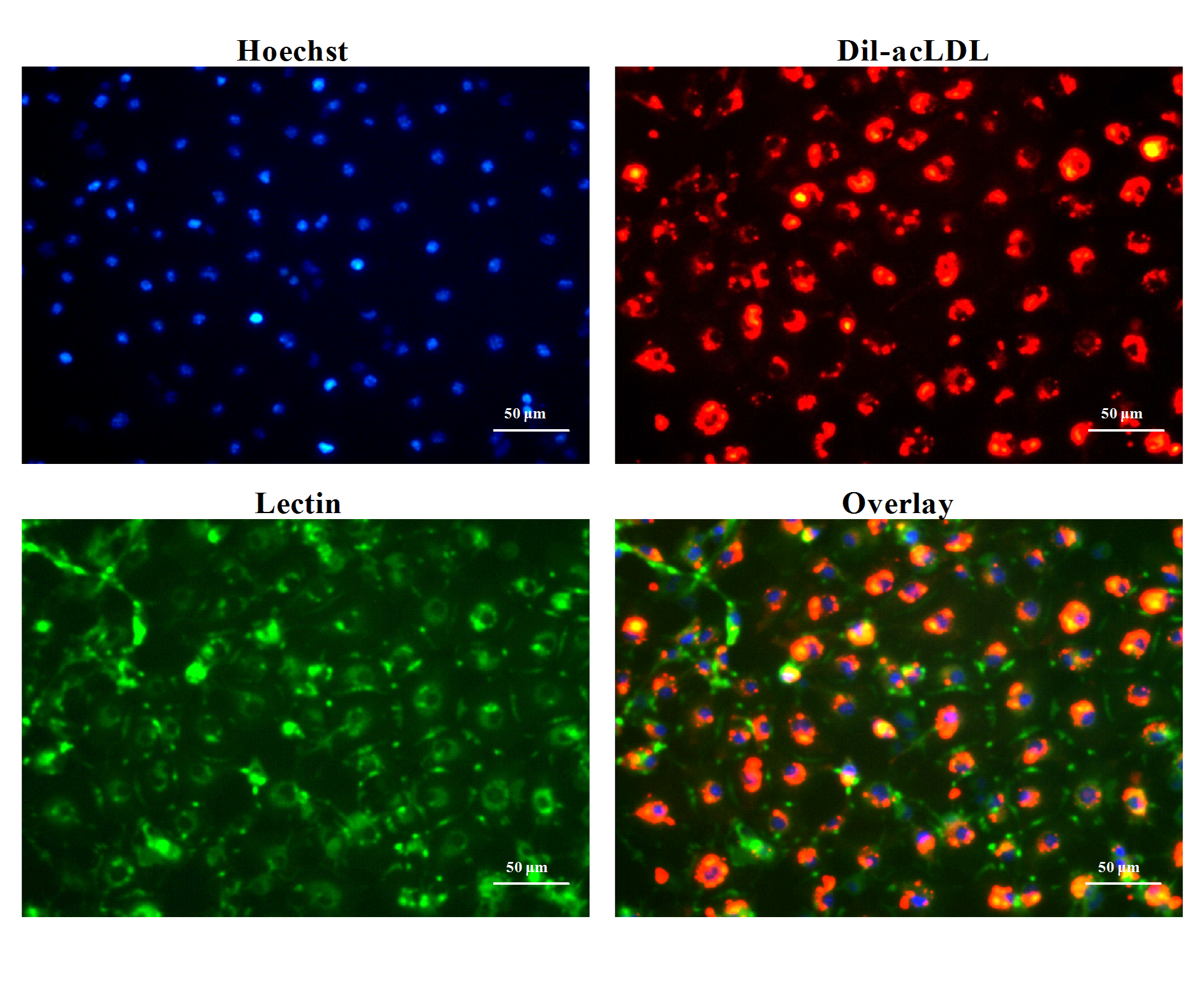

Supplement: Supplementary file 2 — 10.1186/s12933-016-0408-3 Characterization of mouse BM-EPCs. BM-EPCs were identified as Dil-acLDL (red) and lectin (green) double-positive cells under the fluorescence microscope. Nuclei were counterstained with Hoechst (blue). Scale bar: 50 μm. [file 12933_2016_408_MOESM2_ESM.tif]

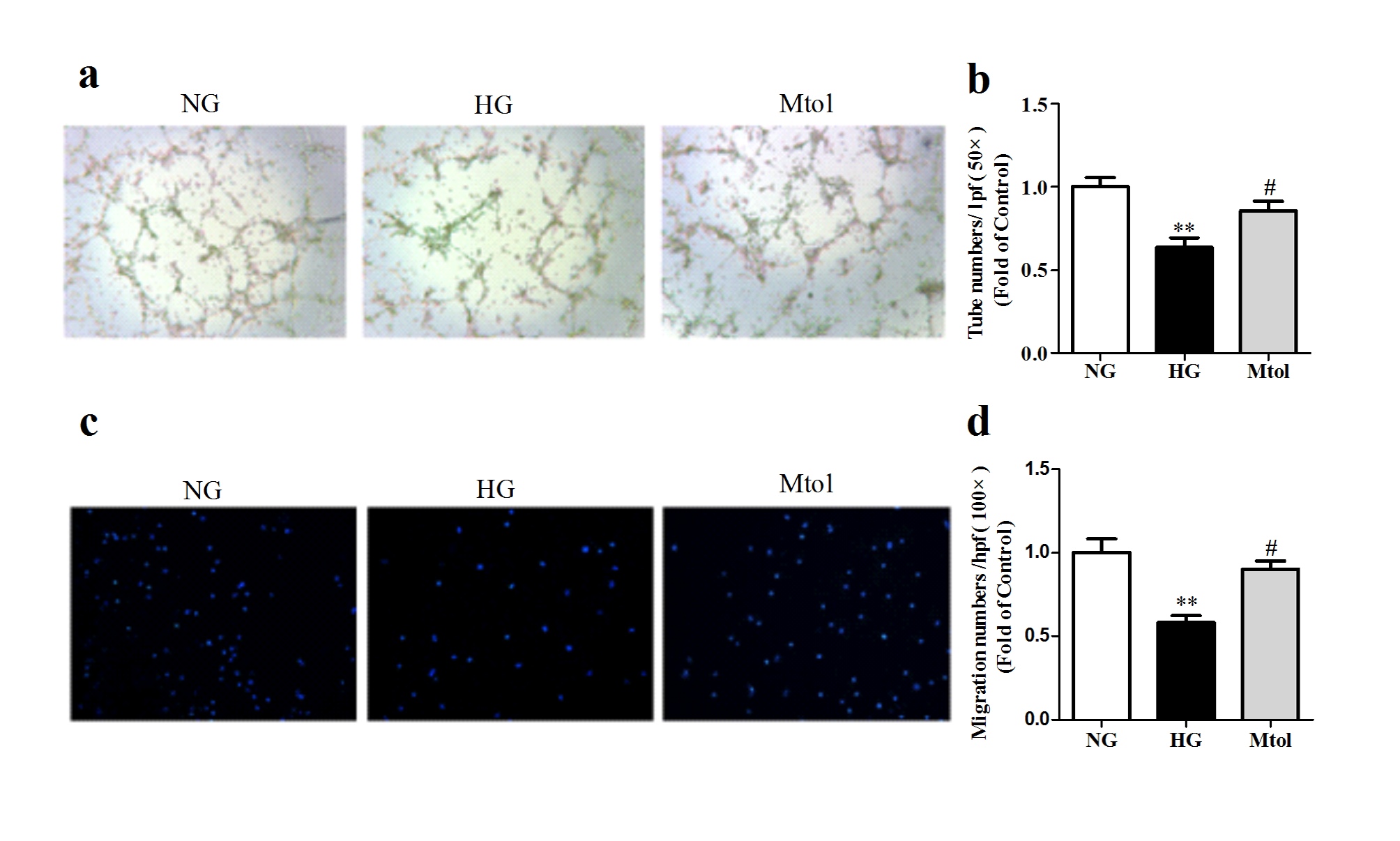

Supplement: Supplementary file 3 — 10.1186/s12933-016-0408-3 BM-EPC functions under the osmotic pressure equal to that of high glucose (HG). Compared with the normal glucose (NG), BM-EPCs treated by mannitol to make equal osmotic pressure with HG showed no significant changes in tube formation and migration.**P < 0.01, vs NG; # P < 0.05 vs HG. Values are mean ± SEM (n = 5 per group). [file 12933_2016_408_MOESM3_ESM.tif]

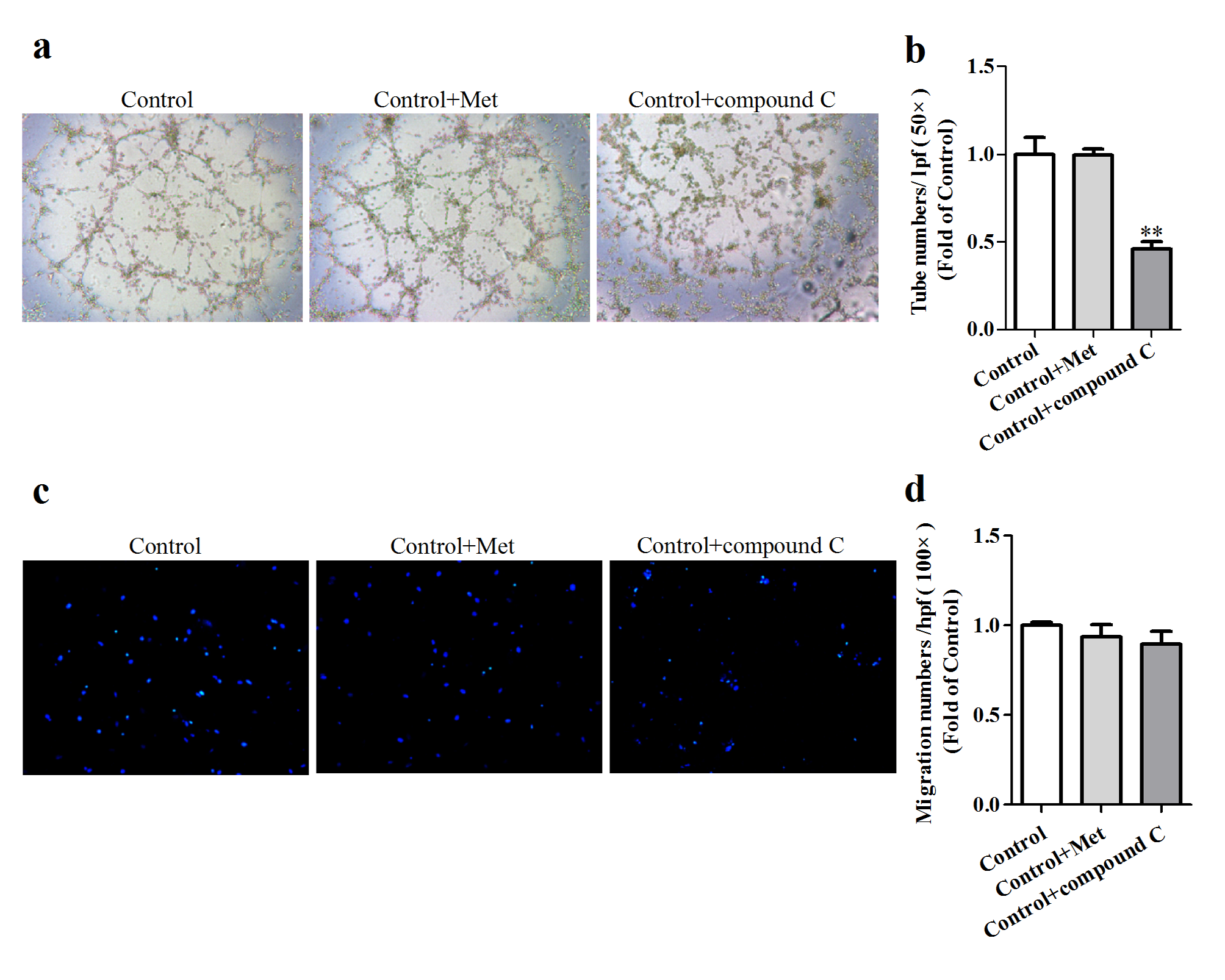

Supplement: Supplementary file 4 — 10.1186/s12933-016-0408-3 Metformin and compound C used alone on BM-EPC functions. Compared with the control, metformin did not change BM-EPC functions of both tube formation and migration. However, compound C significantly inhibited tube formation, but not migration. **P < 0.01, vs Control. Values are mean ± SEM (n = 5 per group). [file 12933_2016_408_MOESM4_ESM.tif]
